# Amdoparvoviruses in small mammals: expanding our understanding of parvovirus diversity, distribution, and pathology

**DOI:** 10.3389/fmicb.2015.01119

**Published:** 2015-10-12

**Authors:** Marta Canuti, Hugh G. Whitney, Andrew S. Lang

**Affiliations:** ^1^Department of Biology, Memorial University of NewfoundlandSt. John’s, NL, Canada; ^2^Animal Health Division, Forestry and Agrifoods AgencySt. John’s, NL, Canada

**Keywords:** amdoparvovirus, aleutian mink disease virus, AMDV, farmed mink, parvovirus, fox viruses, ferret viruses

## Abstract

Many new viruses have been discovered recently, thanks in part to the advent of next-generation sequencing technologies. Among the *Parvoviridae*, three novel members of the genus *Amdoparvovirus* have been described in the last 4 years, expanding this genus that had contained a single species since its discovery, *Aleutian mink disease virus*. The increasing number of molecular and epidemiological studies on these viruses around the world also highlights the growing interest in this genus. Some aspects of amdoparvoviruses have been well characterized, however, many other aspects still need to be elucidated and the most recent reviews on this topic are outdated. We provide here an up-to-date overview of what is known and what still needs to be investigated about these scientifically and clinically relevant animal viruses.

## Aleutian Mink Disease Virus (Amdv) Discovery And Initial Characterizations

Aleutian disease was originally observed in the USA during the late 1940s in Aleutian mink, a novel gunmetal gray variety similar in color to the Aleutian foxes (Gorham et al., 1965). The disease, also called plasmacytosis (a high number of plasma cells in tissues or exudates), was therefore already known to farmers for some years when it was officially reported for the first time in 1956 as a disease of unknown origin (Hartsough and Gorham, 1956). As it initially appeared to be restricted to Aleutian mink it was mistaken for a genetic disorder and often went unnoticed, masked by other outbreaks predominant at that time such as distemper and botulism (Gorham et al., 1965). After devastating plasmacytosis outbreaks following inoculations with infected tissue suspensions used as autologous vaccinations against canine distemper virus, it was realized that an infectious agent was responsible for the disease. Unfortunately the virus had already been shipped worldwide with infected mink (Gorham et al., 1965).

The first scientific evidence of an infectious cause was the demonstration that suspensions of infected mink organs retained their infectivity after filtration, indicating the causative agent was likely viral (Karstad and Pridham, 1962). The virus was subsequently isolated using feline kidney cell lines and it was concluded that it possessed the distinctive properties of a parvovirus (Hahn et al., 1977; Porter et al., 1977). The complete genome was sequenced in 1988 (Bloom et al., 1988).

Fifty years after the discovery of AMDV, three related viruses have been identified in other mammals. AMDV is, however, the best characterized virus in its genus and was the focus of the majority of the literature considered.

## Classification And Relationships Amongst Amdoparvoviruses

Aleutian mink disease virus remained the sole member of the genus *Amdovirus* within the family *Parvoviridae*, subfamily *Parvovirinae*, for many years. It has now been reclassified as *Carnivore amdoparvovirus 1* within the genus *Amdoparvovirus* (ICTVdb, 2014). This genus also contains the other recently discovered *Carnivore amdoparvovirus 2* (gray fox amdovirus, GFAV; Li et al., 2011), the proposed Carnivore amdoparvovirus 3 (raccoon dog and fox amdoparvovirus, RFAV; Shao et al., 2014) and the red fox fecal amdovirus (RFFAV; Bodewes et al., 2014), whose genome has only been partially sequenced and therefore is not yet assigned official taxonomic status.

**FIGURE 1:**
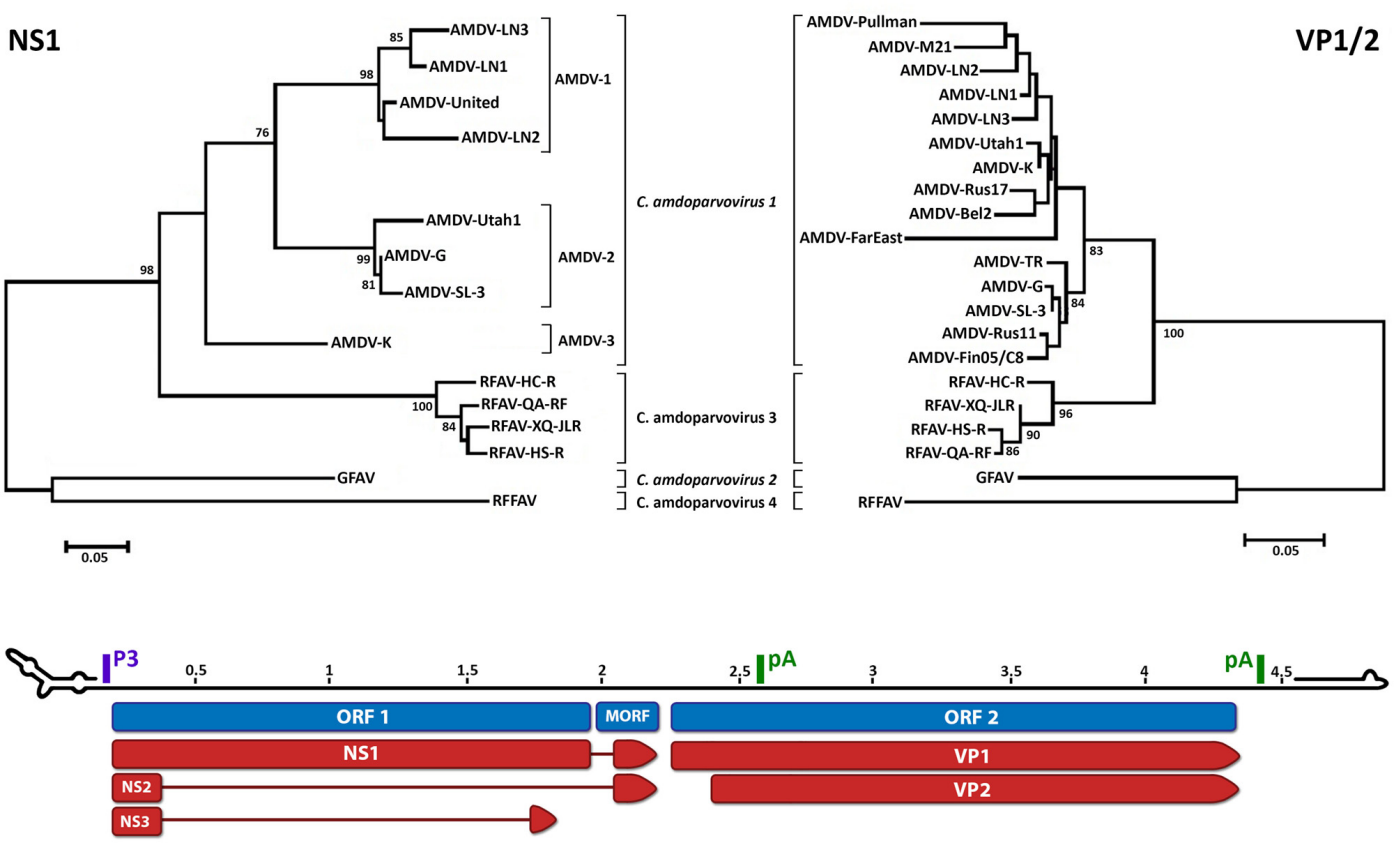
**Phylogenetic analysis and genome organization of amdoparvoviruses.** Analysis based on partial NS1 amino acid sequences is shown in the top-left and based on partial VP1/2 amino acid sequences is shown in the top-right. Species are indicated in italics and proposed species and partially characterized members are not italicized. The three lineages identifiable within *Carnivore amdoparvovirus 1* in the NS1 phylogeny, Aleutian mink disease virus (AMDV)-1 to 3, are indicated. The unrooted trees were built with the Maximum Likelihood method (Felsenstein, 1981) using the best-fitting substitution model identified by modeltest with MEGA6 (Tamura et al., 2013). The NS1 tree is based on alignment of 189 amino acids and the model JTT + G = 1.0973 while the VP1 tree is based on alignment of 274 amino acids and the model JTT + G = 0.4273. Bootstrap values are shown as percentages based on 1000 iterations where support was >75% and the distance bars show the number of substitutions per site. The genome map is shown on the bottom as the plus-sense strand, 5′–3′, with a scale bar in kb. Open reading frames (ORFs) are indicated in blue with the three ORF 1-encoded non-structural proteins, which share the same N-termini but possess unique C-termini, and two ORF 2-encoded capsid proteins indicated in red. The position of the promoter (P3) and polyadenylation sites (pA) are indicated in purple and green, respectively. The predicted structures of the terminal loops are indicated at the two extremities. The right-end terminal palindrome adopts a U-shape configuration with one region of unpaired nucleotides (bubble) and the left-end terminus adopts a Y configuration with a longer arm and two unpaired regions. Accession numbers used to build the NS tree: Aleutian mink disease virus (AMDV)-G, JN040434; ADV-UtahI, X77083; ADV United, X77085; AMDV-SL-3, X97629; ADV-K, X77084; ADV-LN1, GU108231; ADV-LN2, GU108232; ADV-LN3, GU269892; RFAV HC-R, KJ396348; RFAV QA-RF, KJ396349; RFAV XQ-JLR, KJ396350; RFAV HS-R, KJ396347; GFAV, JN202450; red fox fecal amdovirus (RFFAV), KF823809. Accession numbers of additional sequences used to build the VP1 tree: AMDV-Utah1, AMU39015; AMDV-Pullman, U39014; AMDV-K, M63044; AMDV-TR, U39013; AMDV-LN-1, GU183264; AMDV-LN-2, GU183265; AMDV-M21, DQ630722; AMDV-Rus17, KJ174164; AMDV-Bel2, KJ174161; AMDV-FarEast, DQ371395; ADV-Rus11, KJ174158; AMDV-Fin05/C8, GQ336866.

Phylogenetic analysis shows that RFAV is more closely related to AMDV (∼76% amino acid identity for the non-structural protein NS1) and occupies an intermediate position between AMDV and GFAV (**Figure 1**). RFFAV is more closely related to GFAV, with an identity of >80% (Bodewes et al., 2014; Shao et al., 2014). All RAFVs belong to the same species and share ∼95% amino acid identity in NS1, while three distinct lineages can be recognized within *Carnivore amdoparvovirus 1*: AMDV-1, AMDV-2 (within-lineage NS1 identity of 90–94% and 97–99%, respectively) and AMDV-3, which is only 82–84% identical to other AMDVs and might represent a different species (Bloom et al., 1988; Gottschalck et al., 1991, 1994; Schuierer et al., 1997; Li et al., 2012). AMDVs of ferrets appear genetically distinct from those identified in mink (Murakami et al., 2001). Some authors suggest yet more species might exist that have not been sequenced completely (Knuuttila et al., 2015; Persson et al., 2015). Analyses based on the capsid protein sequences reveal different clustering within *Carnivore amdoparvovirus 1*, suggesting the existence of chimeric genomes generated after recombination (Shackelton et al., 2007; Christensen et al., 2011; Li et al., 2012). Clustering on phylogenetic trees does not correlate with pathogenicity or year of sampling but partial geographic grouping can be observed (Knuuttila et al., 2009; Nituch et al., 2012).

## Virus Structure And Protein Expression

Aleutian mink disease virus is a naked, spherical virus of 22–26 nm diameter (Shahrabadi and Cho, 1977; Bloom et al., 1980). Its capsid is characterized by a *T* = 1 icosahedral symmetry and is formed by two different proteins, VP1 and VP2, in an approximate 1:9 ratio for a total of 60 proteins that are capable of self-assembly (Clemens et al., 1992; McKenna et al., 1999; Bloom et al., 2001; Cotmore and Tattersall, 2014). The capsid contains a single molecule of heterotelomeric single-stranded DNA ∼4.8 kb in size (Bloom et al., 1988; Schuierer et al., 1997; Li et al., 2012). Minus-sense strands are encapsidated in preference to plus-sense strands (Best and Bloom, 2006; Cotmore and Tattersall, 2014). As in other parvoviruses NS1 is probably covalently attached to the 5′ end of the genome and is located on the outside of the infectious particles (Cotmore and Tattersall, 1988, 1989; Huang et al., 2014).

The monosense genome consists of two open reading frames (ORFs), flanked by untranslated regions (**Figure 1**). The left ORF encodes the non-structural proteins NS1, NS2, and NS3, and the right ORF encodes the capsid proteins VP1 and VP2 (Clemens et al., 1992; Qiu et al., 2006; Huang et al., 2014). The presence of one or two smaller middle ORFs (MORFs) has been reported for *Carnivore amdoparvovirus 1* and *2* (Bloom et al., 1988; Gottschalck et al., 1991, 1994; Li et al., 2011). Six different mRNAs are generated from a single pre-mRNA, which is transcribed from a single promoter, by alternative splicing and alternative polyadenylation, with two polyadenylation sites present (Best and Bloom, 2006; Qiu et al., 2006; Huang et al., 2014).

The major non-structural protein, NS1, possesses ATPase and helicase activity and ATP- and DNA-binding domains. It localizes to the nucleus and is crucial for viral replication with roles in DNA replication, regulation of transcription, and capsid assembly (Bloom et al., 1988; Christensen et al., 1995; Huang et al., 2014). NS2 co-localizes in the nucleus whereas NS3, produced at lower levels, has extra-nuclear localization. Both proteins are crucial for viral replication (Huang et al., 2014). The capsid protein VP1 contains the entire sequence of VP2, but is ∼40 amino acids longer on the N-terminal side. Both proteins are targets of the immune response (Mori et al., 1991; Bloom et al., 2001). Unlike other parvoviruses, VP1 does not contain a phospholipase A2 (PLA2) domain that mediates virus transfer across the endosomal bilayer during infection (Zádori et al., 2001; Cotmore and Tattersall, 2014).

As in all parvoviruses, amdoparvoviruses possess palindromic sequences at both genomic extremities (**Figure 1**). These fold into hairpin structures and mediate viral DNA replication by functioning as primers and allowing the attachment of host polymerases, and they are also essential for packaging (Cotmore and Tattersall, 2014). AMDV is defined as heterotelomeric because the left and right genomic termini differ in both size and sequence, and therefore in structure (Y- and U-shaped, respectively; Bloom et al., 1988, 1990; Ranz et al., 1989; Willwand and Kaaden, 1990).

## Virus Tropism and Replication

Similar to other parvoviruses, AMDV replication is S-phase-dependent, requiring actively dividing cells to produce new progeny (Cotmore and Tattersall, 1987, 2014; Oleksiewicz and Alexandersen, 1997). In adult mink AMDV infects macrophages and viral entry is probably mediated by cellular Fc receptors recognizing antibody-covered viral particles (Mori et al., 1991; Kanno et al., 1993). Antibodies against the virus enhance viral entry into cells, a phenomenon called antibody-dependent enhancement (ADE; Kanno et al., 1993; Dworak et al., 1997). In mink kits the virus infects alveolar type II cells of the lungs and causes cell death (Alexandersen et al., 1987; Park et al., 2005).

The absence of a PLA2 domain in VP1 might reflect a mechanism for viral release from endosomes different from other parvoviruses (Zádori et al., 2001). Parvovirus NS1 is a cytotoxic protein that induces apoptosis (Hsu et al., 2004), an essential phenomenon for productive AMDV infection. It has been shown that cellular caspases, proteins required during apoptosis (Lamkanfi et al., 2006), cleave NS1 at two different sites (Best et al., 2002, 2003) and the cleaved NS1 is actively transported into the nucleus where genome replication, regulated by NS proteins, occurs (Huang et al., 2014).

Parvoviruses replicate their genome by rolling hairpin replication (RHR), a mechanism adapted from the rolling circle replication used by some circular DNA viruses [reviewed in (Berns, 1990)]. Replication starts when the host polymerase binds to the 3′ terminal hairpin and the genome is converted into double-stranded DNA. NS1 then initiates strand displacement replication, which involves the folding and unfolding of a single DNA molecule repeatedly rearranged into intermediate replication forms (Berns, 1990). Although AMDV DNA replication has not been completely studied, the identification of several replication intermediates is consistent with an RHR model (Bloom et al., 1983; Löchelt et al., 1989). Finally, VP1 of AMDV is able to specifically bind viral DNA when folded into a secondary intermediate replication structure, and this binding seems to be responsible for the simultaneous segregation and encapsidation of the single-stranded genomes into empty capsids (Cotmore and Tattersall, 1987; Willwand and Kaaden, 1990).

## Transmission and Host Range

All bodily fluids (blood, saliva, feces, and urine) can contain viruses and transmission via direct or indirect (contaminated feed, water, or environment) contacts are both possible (Kenyon et al., 1963; Gorham et al., 1964; Pennick et al., 2005; Shao et al., 2014). Virions are highly resistant in the environment and can survive high temperatures and chemical treatments (Eklund et al., 1968; Hahn et al., 1977; Porter et al., 1977). This stability makes the virus difficult to eliminate from contaminated surfaces and facilitates viral spreading in the wild, and within and between farms (Prieto et al., 2014). AMDV can also be transmitted vertically from infected females to kits, with not only trans-placental transmission possible but the placenta also representing a site of viral replication (Broll and Alexandersen, 1996). Finally, a study demonstrated that the mosquito *Aedes fitchii*, which can feed on mink, can carry AMDV for up to 35 days after an infectious blood meal, and the virus may replicate in this vector indicating that vector-borne transmission may also be possible (Shen et al., 1973).

Experimental infection and DNA detection studies showed that AMDV can infect other mustelids besides mink (weasels, badgers, ferrets, otters) and also other animals such as skunks, raccoons, bobcats, cats, dogs, mice, and humans (Porter et al., 1982; Alexandersen et al., 1985; Oie et al., 1996; Mañas et al., 2001; Pennick et al., 2007; Allender et al., 2008; Jepsen et al., 2009; Farid, 2013; Knuuttila et al., 2015). *Carnivore amdoparvovirus 2* has been identified only in gray fox to date (Li et al., 2011), whereas RFAV has been found in Arctic fox and raccoon dog (Shao et al., 2014) and RFFAV has been found only in feces of wild red fox (Bodewes et al., 2014).

## Epidemiology

Aleutian mink disease virus affects farmed and free-living animals, where it propagates and evolves with different dynamics. Viruses identified in the two reservoirs and in different locations can be very similar, indicating exchange of strains between the wild and farms, and amongst different farms following the movement of animals or contaminated materials. Accidental escape or deliberate release of infected animals from farms, where intensive farming facilitates disease spread, makes farms a source of viruses for wild populations (Gunnarsson, 2001; Mañas et al., 2001; Fournier-Chambrillon et al., 2004; Nituch et al., 2011, 2012).

The AMDV epidemic is thought to have originated in North America, which is where mink farming began during the late 1800s. Even though the disease was first described in farmed animals, it is unknown if the virus first emerged in the wild or on farms (Hartsough and Gorham, 1956; Gorham et al., 1965; Nituch et al., 2012). The only available AMDV epidemiological data for North America come from Canada (**Table 1**). In Nova Scotia the virus is highly prevalent in wild mink (up to 93% of animals carry the virus or anti-amdoparvovirus antibodies) and in other wild animals (**Table 1**). It is also prevalent on farms (up to 70% in 2003), but efforts for implementing eradication strategies are reducing the number of affected animals (Farid et al., 2010, 2012; Farid, 2013). During the 1970s a study in Ontario reported a 62% seroprevalence in wild animals and 46% of farms contained seropositive animals (up to 85% per farm), while from 1986–2006 14–60% of farms tested positive and 25–38% of wild animals were positive from 2005–2009 (Cho and Greenfield, 1978; Nituch et al., 2011, 2012).

**Table 1:** Amdoparvovirus epidemiology in wild and farmed animals.

Geographical area	Origin^∗^	Species^§^	Prevalence (%)^&^	Detection method^£^	Period	Study
**Aleutian mink disease virus (AMDV)**						
Nova Scotia, Canada	W	American mink	78.6	PCR	2006–2008	Farid et al., 2010
	W	American mink	93.3	CIEP, PCR	2009–2011	Farid, 2013
	W	Ermine	70.5			
	W	Striped skunk	25			
	W	Northern river otter	18.2			
	W	Northern raccoon	10.6			
	W	Bobcat	10			
	F	American mink	23.8–70.7	CIEP	1998–2005	Farid et al., 2012
Ontario, Canada	W	Mink sp.	61.2	CIEP	Early 1970s	Cho and Greenfield, 1978
	W	Mink sp.	29–38	CIEP	2005–2009	Nituch et al., 2011
	W	Mink sp.	25	PCR	2005–2009	Nituch et al., 2012
	F	Mink sp.	46.3	CIEP	1970s	Cho and Greenfield, 1978
	F	Mink sp.	14–60	/	1986–2006	Nituch et al., 2012
USA	F	Mink sp.	/	PCR	1990s	Hadlow et al., 1983
	F	Mink sp.	/	PCR	1990s	Oie et al., 1996
Spain	W	European mink	33	CIEP, PCR	1997–1999	Mañas et al., 2001
	W	American mink	40	PCR		
	W	Eurasian otter	100^$^	PCR		
UK	W	American mink	51.8	CIEP	2000	Yamaguchi and Macdonald, 2001
	D	Ferret	8.5	Serology	1993	Welchman et al., 1993
France	W	American mink	22.7	CCIE, CCLAIE	1991–2001	Fournier-Chambrillon et al., 2004
	W	European mink	12.1			
	W	European polecat	11			
	W	Stone marten	23.5			
	W	Pine marten	6.3			
	W	Common genet	4.4			
Denmark						
Bornholm island	W	Mink sp.	45.1	CCIE, PCR	1998–2009	Jensen et al., 2012
Mainland	W	Mink sp.	3.3	CCIE		
	F	Mink sp.	5	/	2001	Christensen et al., 2011
Sweden	W	Mink sp.	46,58		2004–2009	Persson et al., 2015
	F	Mink sp.	/	/	2000s	Persson et al., 2015
Finland	W	American mink	54.4	ELISA, PCR	2006–2014	Knuuttila et al., 2015
	W	European badger	26.9			
	W	European polecat	7.1			
	F	Mink sp.	3–60^#^	CIEP	1980–2014	Knuuttila et al., 2015
Estonia	W	American mink	14.8	PCR	2007–2010	Leimann et al., 2015
Ireland	F	Mink sp.	80	PCR	2006	Jahns et al., 2010
Iceland	W	Mink sp.	3.6	CCE	1980s	
	F	Mink sp.	/	CCE	1996	Gunnarsson, 2001
The Netherlands	F	Mink sp.	/	PCR	2005	Christensen et al., 2011
Germany	F	Mink sp.	/	PCR ISH + SB	1980s	Schuierer et al., 1997 Haas et al., 1988
China	F	Mink sp.	/	/	2009	Li et al., 2012
Russia	F	Mink sp.	/	PCR	2000s	Martynenko and Bogunov, 2007
**Red fox fecal amdovirus (RFFAV)**						
Spain	W	Red fox	/	Metagenomics	2013	Bodewes et al., 2014
**Gray fox amdovirus (GFAV)**						
California	F	Gray fox	/	PCR	2009	Li et al., 2011
**Raccoon dog and fox amdoparvovirus (RFAV)**						
China	F	Arctic fox	/	PCR	2012–2013	Shao et al., 2014
	F	Raccoon dog	/	PCR	2012–2013	

*^∗^F, farm; W, wild; D, domestic.*

^§^*American mink*, Neovison vison; *European mink*, Mustela lutreola; *ermine*, Mustela erminea; *striped skunk*, Mephitis mephitis; *northern river otter*, Lontra canadensis; *northern raccoon*, Procyon lotor; *bobcat*, Lynx rufus; *Eurasian otter*, Lutra lutra; *European polecat*, Mustela putorius; *stone marten*, Martes foina; *pine marten*, Martes martes; *common genet*, Genetta genetta; *European badger*, Meles meles; *ferret*, Mustela putorius; *mink sp.*, *species not specified in the study; red fox*, Vulpes vulpes; *gray fox*, Urocyon cinereoargenteus; *Arctic fox*, Vulpes lagopus; *raccoon dog*, Nyctereutes procyonoides.

*^&^Unless otherwise indicated values refer to the prevalence of infected wild animals for W or percentage of affected farms for F. A / indicates that the data is unknown.*

^£^*PCR, polymerase chain reaction; CIEP, counter immunoelectrophoresis; CCIE, counter-current immunoelectrophoresis; CCLAIE, counter-current line absorption immunoelectrophoresis; CCE, counter current electrophoresis; ISH, in situ hybridization; SB, Southern blot. A / indicates that the data is unknown.*

*^$^Only one individual tested.*

*^#^Annual mean seroprevalence of farmed mink.*

American mink were introduced into Europe during the 1920s and 1930s for farming purposes (Macdonald and Harrington, 2003), and this is probably how AMDV was introduced there (Mañas et al., 2001; Fournier-Chambrillon et al., 2004; Knuuttila et al., 2009). Evidence of infection has been found in various free-ranging animals in different European countries (**Table 1**).

Aleutian mink disease virus is also present in European farms (**Table 1**). Between 1980 and 2014 the seroprevalence in Finnish farms fluctuated between 3 and 60%, a recent report indicates that 80% of farms in Ireland have Aleutian disease, and the virus was also reported in German and Dutch farms (Haas et al., 1988; Jahns et al., 2010; Knuuttila et al., 2015). In Denmark, eradication strategies were implemented in 1999 and only 5% of farms were Aleutian disease-positive as of 2001. In Iceland, eradication of the virus was achieved after infection rates up to 90% on affected farms were reported during the 1970s, but the virus was re-introduced during the late 1990s (Skírnisson et al., 1990; Gunnarsson, 2001; Christensen et al., 2011). A serological investigation also demonstrated that 8.5% of domestic ferrets owned by members of a ferret club were amdoparvovirus antibody-positive (Welchman et al., 1993).

Mink were exported to eastern Europe and Asia between the 1930 and 1960s from Finland, Denmark, and North America and the presence of the virus has been reported in Russia and in China, where the seroprevalence was 43–67% in 2010 (Macdonald and Harrington, 2003; Martynenko and Bogunov, 2007; Knuuttila et al., 2009; Li et al., 2012).

Our understanding of the epidemiology of the other amdoparvoviruses is much more limited (**Table 1**). RFAV was identified in six farms in China, RFFAV in wild foxes in the Spanish Basque region, and GFAV in two animals in California (Li et al., 2011; Bodewes et al., 2014; Shao et al., 2014).

## Clinical Signs And Pathogenesis

Infection of mink by AMDV manifests in different ways depending on the age of the mink, with different pathology and cell-tropism in kits versus adults. In adults, the virus causes a persistent infection that leads to a progressive wasting syndrome, characterized by weight loss and anorexia associated with splenomegaly, lymphadenopathy, plasmacytosis, hypergammaglobulinemia, necrotizing arteritis, and proliferative glomerulonephritis (Eklund et al., 1968; Hadlow et al., 1984). The plasmacytosis leads to a massive production of antibodies, which then enhances viral production (Kanno et al., 1993; Dworak et al., 1997) and causes the formation of perivascular and glomerular virus-antibody complexes that deposit in tissues leading to arteritis and glomerulonephritis (Cho and Ingram, 1973; Porter et al., 1973). The role of immunocomplexes in the pathogenesis of AMDV is confirmed by the fact that immunosuppressed mink do not develop lesions when infected (Cheema et al., 1972) and is the reason for vaccination failure (Aasted et al., 1998), although promising results have been obtained with NS1 DNA vaccination (Castelruiz et al., 2005). Finally, the infection reduces pregnancy rates and is associated with decreased litter size, embryonic death and abortion (Broll and Alexandersen, 1996).

In kits the virus causes an acute infection that manifests as respiratory distress and fulminant interstitial pneumonia, which is due to a direct cytopathic effect in pneumocytes, and is mostly fatal within 3 weeks post-infection (Alexandersen, 1986; Alexandersen et al., 1987; Park et al., 2005). Treatment of kits with anti-AMDV antibodies limits viremia, reduces both mortality and severity, and is protective against acute pneumonia but not against the chronic adult form of the disease (Alexandersen et al., 1989). The transfer of maternal antibodies might therefore prevent pneumonia after birth.

In ferrets and skunks, AMDV causes a similar wasting syndrome with kidney impairment and glomerulonephritis (Kenyon et al., 1967; Ohshima et al., 1978; Pennick et al., 2007; Allender et al., 2008). A respiratory disease with severe coughing that leads to hemorrhagic interstitial pneumonia has also been reported in ferrets (Welchman et al., 1993). Neurologic signs and uveitis can also appear in both ferrets and mink and arteritis in the cardiac muscle has been described in ferrets (Hadlow, 1982; Palley et al., 1992; Welchman et al., 1993; Dyer et al., 2000). Human macrophages are susceptible to AMDV infection (Kanno et al., 1993) but infections in humans, clinically similar to the disease in mink, have only been documented in farmers after occupational exposure (Chapman and Jimenez, 1963; Jepsen et al., 2009). They are an exceptionally rare event considering the large number of affected farms and mink.

Asymptomatic and non-persistent infections are common in both mink and ferrets, and in these individuals a lower antibody response and a transient viremia are observed (Larsen and Porter, 1975; An et al., 1978; Hadlow et al., 1984; Pennick et al., 2005). The mortality rate is much higher in mink of the Aleutian genotype compared to non-Aleutian mink (Gorham et al., 1965; Bloom et al., 1975), but the response to infection does not seem genetically determined in mink of the same type (Larsen and Porter, 1975; Hadlow et al., 1983, 1984; Gottschalck et al., 1994). Disease severity also depends on the viral strain. Highly pathogenic viruses cause high mortality in kits and severe disease in all adults, low virulence strains cause low mortality in kits and mild disease in non-Aleutian adults while still causing a severe illness in Aleutian mink, and some lab-adapted strains have lost their pathogenicity (Hadlow et al., 1983; Alexandersen, 1986; Bloom et al., 1988).

Clinical signs reported for RFAV included anorexia, emaciation, growth retardation, thirst, chronic diarrhea, splenomegaly, enlarged lymph nodes, renal cortex congestion, and brittleness in raccoon dog and emaciation, growth retardation, pale swollen kidneys, and severe diarrhea in Arctic fox (Shao et al., 2014). Clinical signs reported for *Carnivore amdoparvovirus 2* in gray fox included gait abnormalities, lymphadenopathy and acute muscle inflammation (Li et al., 2011). Clinical manifestations of RFFAV infection are unknown.

## Concluding Remarks

Amdoparvoviruses cause severe diseases in a wide range of animals, resulting in major economic losses for farmers and threatening wild animal populations. Continued research and an increased awareness of these scientifically and clinically relevant animal viruses will provide a better picture of their total impact as more information becomes available in the coming years and will likely lead to the discovery of additional new species and susceptible hosts.

## Conflict of Interest Statement

The authors declare that the research was conducted in the absence of any commercial or financial relationships that could be construed as a potential conflict of interest.
