# Corrigendum: Amdoparvoviruses in small mammals: expanding our understanding of parvovirus diversity, distribution, and pathology

**DOI:** 10.3389/fmicb.2016.00264

**Published:** 2016-03-01

**Authors:** Marta Canuti, Hugh G. Whitney, Andrew S. Lang

**Affiliations:** ^1^Department of Biology, Memorial University of NewfoundlandSt. John's, NL, Canada; ^2^Animal Health Division, Forestry and Agrifoods AgencySt. John's, NL, Canada

**Keywords:** amdoparvovirus, aleutian mink disease virus, AMDV, farmed mink, parvovirus, fox viruses, ferret viruses

Due to an oversight in our Mini Review article, one funder was incorrectly listed. The Natural Sciences and Engineering Research Council of Canada was incorrectly identified as the National Science and Engineering Research Council of Canada. The correction does not affect the scientific validity of the results.

## Author contributions

All authors listed have made substantial, direct and intellectual contribution to the work, and approved it for publication.

### Conflict of interest statement

The authors declare that the research was conducted in the absence of any commercial or financial relationships that could be construed as a potential conflict of interest.

